# Facing Dementia in Primary Care: A Process Evaluation of a Multicomponent Practice Change Intervention to Improve Dementia Diagnosis in General Practice

**DOI:** 10.3390/ijerph23081086

**Published:** 2026-08-20

**Authors:** Mark Yates, Caroline Gibson, Constance Dimity Pond, Stephanie Daly, Jessica Jebramek, Sharon Daniel, Lyn Phillipson, Kate Laver, Meredith Gresham, Edwin Tan, Henry Brodaty, Jamie Swann, Shahana Ferdousi, Annica Barcenilla-Wong, Nora Wong, Lee-Fay Low

**Affiliations:** 1 Faculty of Health, School of Medicine, Deakin University, Ballarat 3350, Australia; mark.yates@deakin.edu.au (M.Y.);; 2 Wicking Dementia Research and Teaching Centre, University of Tasmania, Hobart 7001, Australia; 3 Australian Centre for Evidence Based Aged Care, Latrobe University, Melbourne 3000, Australia; 4 Faculty of the Arts, Social Sciences and Humanities, University of Wollongong, Wollongong 2500, Australia; 5 College of Nursing and Health Sciences, Flinders University, Adelaide 5000, Australia; 6 Faculty of Medicine and Health, School of Pharmacy, University of Sydney, Sydney 2000, Australia; 7 Centre for Healthy Brain and Aging, University of New South Wales, Sydney, 2000, Australia; 8 School of Health and Social Development, Health Services and Digital Innovation, Deakin University, Burwood 3125, Australia; 9 Translational Health Research Institute, Western Sydney University, Sydney 2000, Australia; 10 Faculty of Medicine and Health, University of Sydney, Sydney 2000, Australialee-fay.low@sydney.edu.au (L.-F.L.)

**Keywords:** dementia, primary care, general practice, implementation science, process evaluation

## Abstract

**Highlights:**

**Public health relevance—How does this work relate to a public health issue?**

- Dementia remains substantially underdiagnosed in primary care, delaying access to treatment, support and care planning.
- Improving dementia diagnosis is a public health priority as population ageing increases the burden of dementia worldwide.

**Public health significance—Why is this work of significance to public health?**

- This study evaluates the implementation of a co-designed, multicomponent intervention to improve dementia diagnosis in Australian general practice.
- The findings provide practical lessons for implementing complex practice-change interventions in routine primary care.

**Public health implications—What are the key implications or messages for practitioners, policy makers and/or researchers in public health?**

- Education alone is insufficient; sustainable practice change requires organizational readiness, implementation support and supportive policy settings.
- These findings can inform implementation of complex primary care interventions beyond dementia.

**Abstract:**

Dementia is a leading cause of disability and death worldwide, yet diagnosis in primary care remains substantially lower than expected, delaying access to treatment, support and future care planning. The Facing Dementia Together Practice Change Program was developed as a co-designed, multicomponent intervention to improve dementia diagnosis in Australian general practice. A mixed-methods process evaluation used the RE-AIM framework with the addition of Appropriateness. The intervention included Primary Health Network (PHN) engagement, GP education, clinical resources, audit and benchmarking reports, specialist support, and a dementia risk-alert tool. Data were collected through surveys, stakeholder interviews, website analytics and program documentation. Clinicians who participated reported increased confidence and changes in dementia-related clinical behaviours, while education, resources and benchmarking were perceived as valuable. However, overall reach was limited by competing clinical priorities, workforce pressures, lack of financial incentives, and PHN implementation challenges. The dementia risk-alert tool was feasible but achieved limited uptake because of software compatibility, usability concerns and incomplete electronic medical record data. Although the program was acceptable to participating clinicians, limited reach constrained its potential impact. These findings highlight organizational, workforce, digital infrastructure and policy factors that influenced implementation of multicomponent interventions in routine primary care.

## 1. Introduction

Dementia is a major global public health challenge, with prevalence projected to increase substantially as populations age [1]. Timely diagnosis enables people living with dementia and their families to access appropriate treatment, support services, future care planning and interventions that may delay functional decline and institutionalization [2]. Despite these benefits, dementia remains substantially underdiagnosed in many countries. In Australia, dementia became the leading cause of death in 2023 [3], yet documented dementia diagnoses in primary care are estimated to be 52% lower than expected based on population prevalence and 32% lower than self-reported diagnoses recorded in the Australian Census [4]. Similar evidence of underdiagnosis has been reported internationally, including the United States [5] and Europe [6], highlighting that improving timely dementia diagnosis remains a global public health priority.

General practitioners (GPs) are central to improving dementia diagnosis because they are often the first health professionals to identify cognitive concerns and coordinate ongoing care [7]. In Australia, clinical practice guidelines recommend specialist involvement in diagnosing dementia where appropriate [8]; however, in routine practice, GPs frequently diagnose and manage dementia [9], particularly where access to geriatricians and neurologists is limited [10]. The expanding evidence for dementia risk reduction and the emergence of blood-based biomarkers are expected to further strengthen the role of primary care in dementia prevention, diagnosis and ongoing management [11]. Ensuring that general practice is equipped to undertake these roles is therefore increasingly important.

Despite substantial investment in dementia education, achieving sustained changes in primary care practice has proven difficult. Australia has provided nationally coordinated dementia education programs for GPs [12] and general practice nurses (GPNs) [13] for more than a decade. Although these initiatives improved clinician knowledge and confidence, there is little evidence that education alone results in sustained changes in clinical practice [14]. Similarly, systematic reviews conclude that educational interventions are more effective when combined with organizational, behavioural and system-level implementation strategies [15,16]. Qualitative studies undertaken with people living with dementia, carers and other stakeholders continue to report that GPs dismiss concerns about cognitive decline [17], are reluctant to diagnose dementia, and often prefer to refer to specialists [18,19]. Research with primary care clinicians identify barriers including limited consultation time, competing clinical priorities, perceptions that diagnosis offers limited benefit, inadequate remuneration for cognitive assessment, uncertainty regarding diagnosis, stigma surrounding dementia, and poor access to specialist and community support services [9,18,19].

These findings informed the development of the Facing Dementia Together Practice Change Program (henceforth referred to as the Practice Change Program), a co-designed, multicomponent intervention developed to address barriers operating at clinician and practice levels. The intervention was informed by the COM-B model of behaviour change and incorporated complementary strategies including clinician education, practice resources, audit and benchmarking, digital decision support and implementation support through Australia’s Primary Health Networks (PHNs) [20]. PHNs are federally funded regional organizations responsible for supporting quality improvement and service coordination within Australian general practice [21].

Because the Practice Change Program comprised multiple interacting components delivered within clinical practices, understanding implementation is essential because successful implementation depends not only on intervention design but also on the environments in which interventions are delivered [22]. Process evaluations complement outcome evaluations by examining how interventions are delivered, how participants engage with intervention components, and how organizational and contextual factors influence implementation and outcomes [23].

This study reports the process evaluation of the Practice Change Program using a modified RE-AIM framework [24] with the addition of Appropriateness from Proctor framework [25]. RE-AIM, with the addition of Appropriateness from Proctor’s framework [25], was used to structure examination of intervention implementation, reach, clinician-reported outcomes, uptake, and sustainability, together with the contextual factors influencing delivery.

The intervention was implemented across two PHN regions—WentWest Sydney, serving metropolitan Western Sydney, and Western Victoria PHN, serving predominantly regional and rural communities—providing an opportunity to evaluate implementation across diverse primary care contexts.

A concurrent community awareness campaign was undertaken within the WentWest PHN region to encourage help-seeking for dementia diagnosis; however, this campaign did not involve direct engagement with general practice, it is and is reported separately [17].

The aim of this study was to examine the implementation of the Practice Change Program and the contextual factors influencing its delivery and uptake, using RE-AIM with the addition of Appropriateness to structure the evaluation.

## 2. Methods

### 2.1. The Intervention

Active implementation of the Practice Change Program occurred from January to September 2024. The timing and duration of exposure varied by intervention component: education and specialist-support activities were delivered at scheduled time points, while online Practice Change Program resources remained publicly available beyond the active implementation period. Dementia Training Australia continued to offer dementia education through its ongoing national program. Process-evaluation data were collected during active implementation and for three months following its completion.

The Practice Change Program was implemented between January and September 2024. The intervention has been described in detail elsewhere [20]. Briefly, it was developed as a co-designed, multicomponent practice change intervention informed by behaviour change theory to address barriers to timely dementia diagnosis operating at clinician and practice levels.

The intervention comprised four complementary components: (1) practice engagement and promotion; (2) GP dementia education, including online education, in-practice education sessions and specialist geriatrician support; (3) clinical practice resources, including dementia identification and management checklists, cognitive assessment and diagnostic decision-support tools, and educational videos describing practice team roles; and (4) practice-change drivers, comprising tailored audit and benchmarking reports and a dementia risk-alert tool integrated into compatible electronic medical record (EMR) systems. During implementation, supplementary face-to-face and lunchtime in-practice education sessions were introduced to increase clinician engagement.

Implementation was undertaken in partnership with WentWest Sydney PHN, serving metropolitan Western Sydney, and Western Victoria PHN, serving predominantly regional and rural communities. They were selected as implementation partners because of their established relationships with general practices and recognized role in supporting practice improvement activities.

To support implementation, PHN practice facilitators completed a one-hour training session delivered by members of the research team before commencement of the program. The training covered dementia epidemiology, the rationale and components of the intervention, implementation strategies, available resources and the respective roles of the PHNs and research team in supporting program delivery.

### 2.2. Study Design

A mixed-methods process evaluation was undertaken using the modified RE-AIM framework (Reach, Effectiveness, Adoption, Implementation and Maintenance) together with Appropriateness from Proctor’s implementation outcomes framework [24,25]. RE-AIM provides a structured approach for evaluating implementation across multiple domains relevant to complex interventions, while Appropriateness captures the perceived relevance, usefulness and suitability of intervention components. RE-AIM was used to structure the process evaluation, with the nature and strength of available evidence varying across domains according to the quantitative and qualitative data available.

**Figure 1:**
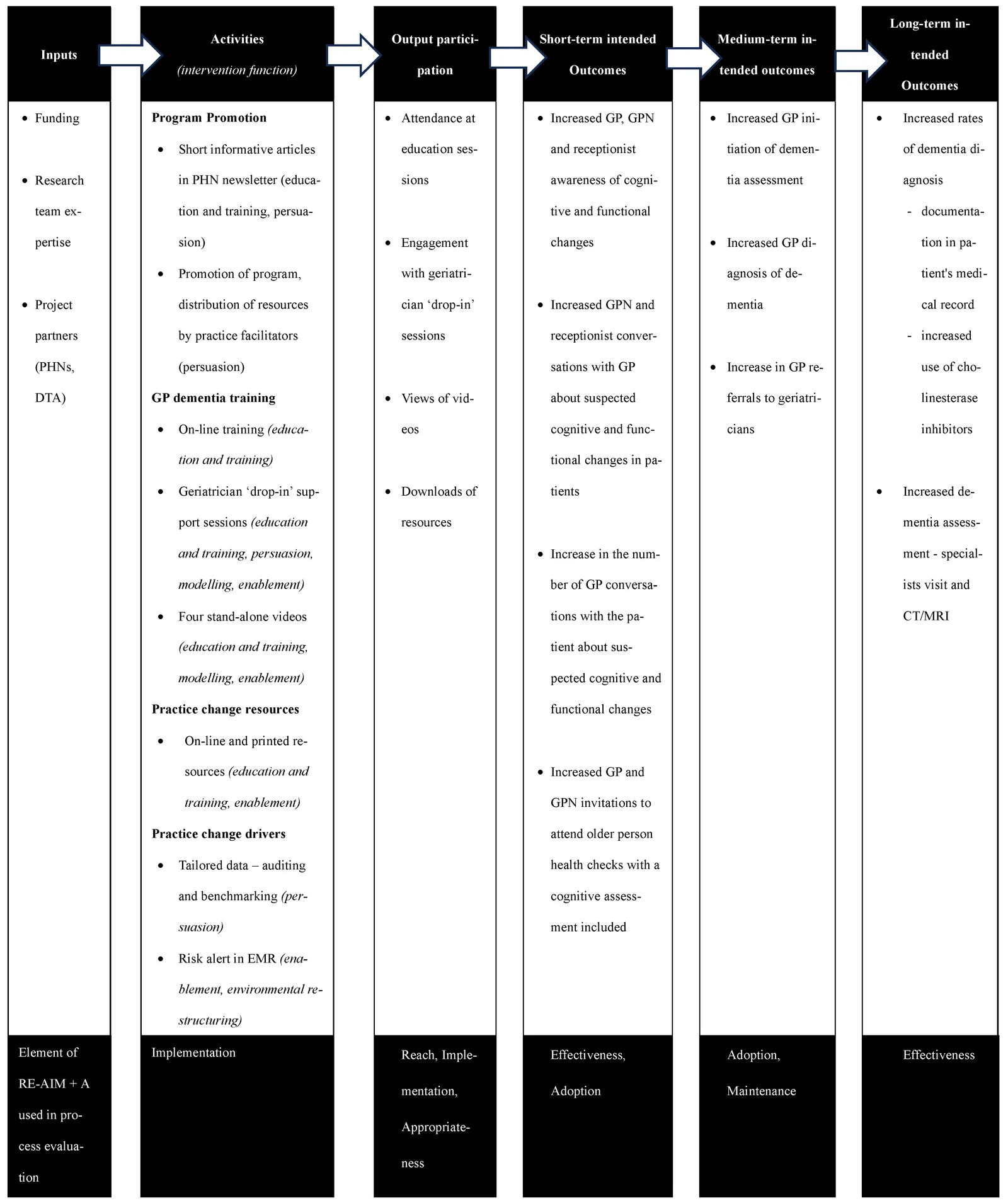
Logic model of the Facing Dementia Practice Change Program showing intervention components from RE-AIM + Appropriateness (A) evaluation domains and intended outcomes. Not all intended outcomes or RE-AIM domains could be fully assessed within the available process evaluation data.

The evaluation used multiple quantitative and qualitative data sources. Figure 1 presents the intervention logic model and evaluation framework, while Table 1 summarizes the evaluation domains and associated data sources.

The reporting of the study followed the Standards for Reporting Qualitative Research (SRQR) [26] (see Table S1).

### 2.3. Study Participants

Participants included PHN staff, Dementia Training Australia GP educators, GPs, GPNS and other stakeholders involved in implementing or participating in the Practice Change Program. Recruitment methods differed according to each data collection activity and are described below. Clinicians who participated in Practice Change Program activities were invited to contribute to the process evaluation. To capture perspectives on barriers to engagement, purposive recruitment also targeted GPs who had been exposed to program promotion but had not participated in intervention activities. Participation was voluntary and no reimbursement was provided to participate in data collection activities.

### 2.4. Program Documentation

Implementation records documented promotional activities, implementation milestones, training activities, participant registrations and attendance, resource distribution, and implementation adaptations.

### 2.5. Website Analytics

Google Analytics was used to measure website engagement, including unique users, resource downloads and educational video views.

### 2.6. Surveys

Immediately following education sessions, participating clinicians were invited to complete an anonymous five-minute pen-and-paper or online survey assessing confidence, knowledge, communication skills, motivation and perceived usefulness of the education.

Participants who consented to follow-up were invited to complete an online survey two months later. The 10 min survey collected brief demographic information and asked participants which Practice Change Program activities they engaged with, and about changes in their knowledge, beliefs and practices as a result of their engagement with the Practice Change Program.

All attendees of the online and face-to-face education sessions were invited to provide contact details to receive a follow-up survey two months after the final training session. Sixteen participants consented to direct follow-up and were subsequently sent an invitation to complete the online survey. In addition, participating PHNs distributed a further survey invitation by email to all education attendees. The 10 min survey collected brief demographic information and asked participants which Practice Change Program activities they had engaged with and about perceived changes in their knowledge, beliefs and clinical practices following engagement with the program.

### 2.7. Stakeholder Interviews

Purposeful and convenience sampling were used to recruit stakeholders involved in the development, implementation and delivery of the Practice Change Program. Participants were invited by email to participate in a 45 min online interview or focus group. Semi-structured interviews and one focus group were conducted with PHN managers, practice facilitators, Dementia Training Australia GP educators, GPs and GPNs who participated in the program. Purposeful sampling was also used to recruit GPs who did not engage with the intervention to better understand barriers to participation. Interview questions explored participants’ experiences with the Practice Change Program, implementation successes and challenges, perceived achievements, contextual influences, and recommendations for future implementation.

### 2.8. Meeting Records

Minutes from Practice Change Program implementation meetings were reviewed to document implementation decisions, adaptations and contextual issues encountered during delivery.

### 2.9. Data Analysis

Quantitative survey data were analyzed descriptively using IBM SPSS Statistics 20.0. Participant characteristics for the two-month follow-up survey were summarized descriptively. Immediate post-training surveys were completed anonymously. Survey results are presented as responses (numerator)/number responding to each question (denominator), with missing data identified where applicable.

Interview and focus-group transcripts were analyzed using manifest content analysis in NVivo Version 14. A deductive coding framework was developed a priori based on the RE-AIM domains and Appropriateness, reflecting the process-evaluation questions. ShD coded all transcripts, with ABW independently reviewing the coding across all transcripts. The coding framework and interpretation of findings were discussed and refined by LFL, MY and CG during analysis. Differences in coding or interpretation were resolved through discussion and consensus.

Quantitative and qualitative data were analyzed separately and subsequently integrated during mixed-methods interpretation. Findings were compared within and across RE-AIM domains to identify convergence, divergence and contextual explanations across survey, interview and implementation data. Data sources were not quantitatively combined or weighted, and interpretation reflected the nature and amount of evidence available within each domain.

Formal assessment of thematic saturation was not undertaken. Instead, sampling sought to achieve data sufficiency by capturing perspectives across the stakeholder groups relevant to the process evaluation.

### 2.10. Reflexivity and Trustworthiness

The researchers are a multidisciplinary team with experience in primary care clinical dementia care, complex intervention design and/or dementia research. MY (MBBS, geriatrician, male); CG (PhD, primary care nurse, female), CDP (PhD, general practitioner, female); SD (general practitioner, female); JJ (health researcher, female); LP (PhD, public health researcher, female); KL (PhD, occupational therapist, female); MG (PhD, occupational therapist, female); ET (PhD, pharmacist, male); HB (dementia researcher, psychiatrist, male); JS (PHN health analytics researcher, male); SF (health researcher, female), ShD (PhD, health researcher, male), ABW (PhD, health researcher, female), NW (health researcher, female) and LFL (PhD, psychologist, female). Throughout analysis, researchers engaged in regular discussions to reflect on how their professional backgrounds and assumptions may have influenced interpretation of the findings.

Trustworthiness was strengthened through data triangulation, maintenance of an audit trail documenting analytical decisions, and the use of participant quotations to support interpretation.

## 3. Results

### 3.1. Participant Characteristics

Participant characteristics are summarized in Table 2. Immediate post-training/activity surveys were completed by 126 of 277 online education attendees (45%) and 16 of 16 face-to-face education attendees (100%). Three clinicians completed the two-month follow-up survey. Twenty-four stakeholders participated in interviews or focus groups, comprising PHN staff, clinicians who engaged with the program, and Dementia Training Australia GP trainers. Despite purposive recruitment of GPs who had not engaged with the intervention, only one non-participating GP was recruited for interview.

### 3.2. Implementation

#### 3.2.1. Program Delivery

The Practice Change Program was delivered largely as intended between January and September 2024. All planned intervention components—including promotion, clinician education, resources, audit and benchmarking reports, and the dementia risk-alert tool—were implemented. However, several components required modification during delivery because of contextual and organizational challenges.

##### Promotion and Engagement

Promotion of the Practice Change Program was undertaken by both participating PHNs using newsletters, practice facilitator visits, meetings with practices and distribution of educational resources. Practice facilitators reported that these resources supported engagement with practices. However, program delivery was constrained by staff turnover, competing organizational priorities, limited implementation capacity and uncertainty regarding PHN roles and responsibilities.

*“We have a contract from the Department of Health with priorities we must achieve under that contract”* (Participant 2, Western Victoria PHN) and *“[the project] wasn’t allocated the resources as a declared project within the PHN itself… it was not built into the PHN budget with dedicated staff.”*

(Practice Facilitator, WentWest PHN)

Participants also described difficulties engaging directly with GPs, as PHN interactions more commonly occurred with practice nurses and practice managers. Communication challenges between the research team and PHNs, together with differing expectations of the PHN implementation role, further reduced implementation consistency.

#### 3.2.2. GP Education

The three rounds of 90 min on-line live dementia training targeting GPs and GPNs was implemented as planned. Round one occurred in February/March 2024, round two in April/June 2024 and round three in August/September 2024.

Although interest in the program was high, attendance was substantially lower than registrations. PHN staff and clinicians consistently described competing clinical priorities, limited time and workforce pressures as major barriers to participation.

*“One of the biggest challenges, and this is not specific just to the dementia training, was that move from interest and registration to be involved in it … very often we find that attendance is low at training sessions and so on, because people get caught up with patients, or they’ve just done a 12 h day. You’re not necessarily just targeting someone that only deals with people with dementia.”*

(Practice Change Facilitator, Went West PHN)

To improve engagement during implementation, additional strategies were introduced, including:

- One face-to-face education session in Went West PHN;
- Lunchtime education delivered within West Vic PHN;
- Additional PHN promotion;
- Direct communication from a local geriatrician.

These adaptations increased opportunities for participation but did not fully overcome engagement challenges.

#### 3.2.3. Clinical Resources

Clinical resources, including dementia assessment checklists, diagnostic support tools and educational videos, were informed by co-design [20] and developed by the research team. The resources were distributed as planned.

PHN staff reported that the resources supported discussions with practices.

*“The provision of resources was amazing… we took it out to practices. You know, having all of that provided to us worked really well to engage people.”*.

(Practice Facilitator, Went West PHN)

Suggestions for improvement included greater integration of templates into existing clinical software. There was feedback from the PHN that the resources could be made more useful to GPs.

*“The only thing, I think to consider with the toolkit is having templates that can be imported into their clinical management system and completed electronically … because it’s time saving for the GP to have that integration into the clinical system.”*

(Primary Care Development Manager, Western Victoria PHN)

### 3.3. Practice-Change Drivers

#### 3.3.1. Dementia Risk-Alert Tool

The dementia risk-alert tool was implemented only within the Western Victoria PHN as compatible software was unavailable in Went West PHN. The planned algorithm, based on the CogDrisk Dementia Risk Assessment Tool [27] could not be fully implemented because several required risk variables were unavailable within routinely collected electronic medical record (EMR) data. Consequently, the algorithm relied on a reduced set of variables, resulting in lower specificity and substantially more patients being identified as potentially eligible for dementia assessment than originally intended.

Using the developed risk alert algorithm, 91,948 patients were identified who met the predefined criteria for a dementia risk assessment and were therefore eligible to receive an alert. Despite this large eligible population, uptake of the tool was limited. During the intervention period, only 34 dementia risk alerts were triggered during consultations, resulting in 76 accesses to the online dementia resources.

Participating GPs also reported concerns regarding software compatibility, usability and confidence in the accuracy of the alerts. Together, these findings indicate that refinement of both the risk algorithm and its integration into clinical software will be necessary before broader implementation.

#### 3.3.2. Audit and Benchmarking

Practice-level audit and benchmarking reports were distributed to 457 general practices across the two PHNs. The reports compared documented dementia diagnoses with expected prevalence based on Australian Institute of Health and Welfare estimates.

### 3.4. Reach

Despite extensive promotion, clinician participation was lower than anticipated. Broad organizational reach was achieved (benchmarking reports and resource dissemination), whereas active clinician engagement (training, specialist support and decision-support tools) was considerably lower.

Interviews suggested that competing clinical priorities, workforce shortages, time pressures and limited financial incentives were the principal barriers to participation. Table 3 describes Reach of the Practice Change Program.

*“One of the biggest challenges, and this is not specific just to the dementia training, was that move from interest and registration to be involved in it … very often we find that attendance is low at training sessions and so on, because people get caught up with patients, or they’ve just done a 12 h day. You’re not necessarily just targeting someone that only deals with people with dementia. And that’s so, it’s competing priorities as well.”*

(Practice Change Facilitator, Went West PHN)

**Table 3:** Reach of the Face Dementia Practice Change Program according to the RE-AIM framework.

Intervention Component	Planned Reach	Actual Reach	Achievement	Interpretation
General practice recruitment	138 practices (95 WentWest; 43 Western Victoria)	Promotion reached all 557 GP practices across both PHNs through newsletters, facilitator visits, benchmarking reports and promotional materials.	Promotion exceeded target, but active participation was substantially lower.	Dissemination through promotion was broad; engagement of practices in intervention activities was the major challenge rather than awareness.
GP dementia training	473 GPs (estimated from target practices)	978 registrations; 277 attendees; 172 GPs (62% of attendees).	172 GPs attended (36% of target).	Training generated considerable interest, but attendance was limited by competing clinical priorities despite CPD accreditation.
Supplementary face-to-face education	Not originally planned.	WentWest: 18 attendees (8 GPs). Western Victoria: education delivered in six practices, reaching 50 GPs, 12 practice nurses and one practice manager.	Additional strategy implemented to improve GP reach.	Practice-based education (West Vic) appeared more successful than the one off in person training event (Went West) for engaging GPs.
Geriatrician support	Regular access to specialist advice for GPs.	Eighteen virtual drop-in sessions were delivered; one GP participated. No further GPs engaged after changing to individual appointments.	Minimal utilization.	Availability of specialist support alone was insufficient to increase GP engagement.
Practice resources	Resources available to participating practices.	270 printed resource packs distributed; 1996 webpage views; 260 resource downloads; 261 educational video views.	Moderate digital engagement.	Resources achieved substantially greater reach than education and support.
Tailored benchmarking reports	Provide dementia diagnosis benchmarking to participating practices.	Tailored benchmarking reports were distributed to 457 GP clinics comparing documented dementia diagnoses with expected prevalence.	High organizational reach.	Benchmarking successfully reached most practices across both PHNs and provided population-specific feedback.
Dementia risk alert	Point-of-care prompts to support earlier dementia assessment.	91,948 eligible patients identified; 34 alerts triggered; 76 accesses to online dementia resources.	Very low implementation reach.	Technical limitations and reduced algorithm specificity resulted in minimal activation during routine consultations.

### 3.5. Appropriateness

Clinicians who participated described the intervention as relevant and useful.

#### Education

Participants valued the practical focus of the education sessions, particularly strategies for:

- Discussing dementia;
- Differentiating mild cognitive impairment from dementia;
- Involving family members;
- Managing driving conversations.

*“I like the very practical tips that were in the webinar … How do we navigate this conversation? Who do we get in the room? How do we approach that conversation tips around driver’s license, which is a very thorny conversation at the best of times, much less when you’re also discussing a very challenging long-term prognosis. So that certainly was useful.”*

(GP, Went West PHN)

### 3.6. Resources

The clinical resources were perceived as practical and applicable to routine clinical practice. Participating clinicians described the assessment checklists and decision-support tools as particularly useful in structuring dementia assessment and management.

*“I was looking for tools for me to work with, which might facilitate management better… the dementia checklist was good… things that could be done once we had broached the subject, that things are a bit of an issue, and how do we further it? And the sorts of questions that you ask.”*

(GP, Western Victoria PHN)

### 3.7. Risk-Alert Tool

Although clinicians recognized the potential value of electronic decision support, most considered the current tool insufficiently integrated into routine workflows and questioned both its usability and accuracy.

*“The risk assessment tool… it doesn’t work with [our medical software] so I don’t get any pop ups … I can access data, but I find it incredibly clumsy and clunky and unreliable … I have not had good experiences with [risk alert tools] … It’s not useful, in my opinion. It is not a useful database, and so we haven’t gone case finding.”*

(GP, Western Victoria PHN)

### 3.8. Audit and Benchmarking

Clinicians consistently reported that these data highlighted previously unrecognized gaps between recorded and expected dementia diagnoses and prompted reflection on dementia identification within their practices.

*“I remember being surprised that our numbers didn’t appear accurate.”*

(General Practitioner, Western Victoria PHN)

### 3.9. Effectiveness: Clinician-Reported Capability and Perceived Behaviour Change

Participating clinicians reported improvements in perceived capability and motivation following engagement with the intervention.

Following education, participants reported:

- Greater confidence diagnosing dementia;
- Improved communication skills;
- Increased confidence in care planning;
- Stronger belief in the value of timely diagnosis.

Interview participants also described increased awareness of the benefits of diagnosis for patients and families.

*“some GPs, I think, have had that sort of fatalistic attitude … because it’s a progressive disease that I can’t do much about, but actually that the positives of getting a diagnosis are really important. I think that’s a big highlight for me … to get that understanding of why it is important and the positives that come out of that.”*.

(GP, Went West PHN)

### 3.10. Adoption: Uptake of Intervention Components and Reported Clinical Behaviours

Evidence relating to Adoption comprised observed uptake of intervention components from implementation records and analytics, together with clinician-reported changes in clinical behaviours. Uptake varied substantially across intervention components, as reported under Implementation and Reach.

Participants reported several changes to clinical practice following the intervention, including:

- Earlier conversations about cognitive change;
- Improved documentation of cognitive concerns;
- Greater involvement of family members;
- Increased collaboration with practice nurses and reception staff;
- Establishment of peer discussions about dementia within practices.

*“And since this [training] we have started an informal lunchtime meeting with other doctors that work in aged care just to sort of have that chance to speak to other colleagues and, etc. and talk about difficult cases. And there’s a Whatsapp group as well. So that’s been really positive. That’s come out of this.”*

(GP, Western Victoria PHN)

These qualitative findings provide examples of perceived behaviour change among participating clinicians but do not establish the extent to which these behaviours were adopted across the broader target population.

### 3.11. Maintenance

Maintenance could not be adequately assessed because only three clinicians completed the two-month follow-up survey. Interview data provided examples of continued use of dementia assessment tools, incorporation of cognitive screening into older-person health assessments and ongoing discussions about dementia within practice teams among some participating clinicians. However, these findings cannot establish sustained implementation or maintenance of practice change across the broader target population.

## 4. Discussion

This mixed-methods process evaluation examined the implementation of the Practice Change Program, a co-designed multicomponent intervention designed to improve dementia diagnosis in Australian general practice. Overall, the intervention was delivered largely as intended, and clinicians who participated reported increased confidence, knowledge and motivation to diagnose and manage dementia. Training, practical resources and tailored audit and benchmarking data were perceived as relevant and useful. However, despite high acceptability among participating clinicians, the overall reach of the intervention was lower than anticipated. Organizational constraints, workforce pressures, competing clinical priorities, digital infrastructure limitations and implementation partnership challenges substantially influenced delivery and reduced the potential impact of the program. These findings reinforce the importance of evaluating implementation processes alongside intervention outcomes to understand not only whether complex interventions are delivered, but also how contextual factors influence implementation success in routine primary care.

The strength of evidence varied across RE-AIM domains. Evidence was strongest for intervention delivery, reach and contextual influences on implementation, whereas limited longitudinal follow-up constrained assessment of clinician-level Adoption and prevented adequate assessment of Maintenance.

### 4.1. Engagement and Implementation Challenges in Primary Care

Limited participation was one of the principal findings of this process evaluation and reflects the broader challenges reported in Australian and international primary care practice-change initiatives [28,29,30,31,32]. General practitioners increasingly manage older populations with multimorbidity within resource-constrained health systems, where heavy clinical workloads, competing priorities, administrative demands and limited protected time reduce capacity to engage in quality improvement and professional development activities [33,34]. Although continuing professional development points were available, participants consistently identified lack of remuneration as an additional barrier to participation. Together, these findings suggest that willingness to engage is insufficient when organizational and system-level demands limit clinicians’ capacity to participate. This is consistent with the implementation science literature demonstrating that adoption of evidence-based interventions is strongly influenced by organizational context, leadership support, workforce capacity and competing demands, even when clinicians perceive interventions to be useful and relevant [35,36].

An additional structural barrier was the absence of dedicated Australian Government primary care funding (Medicare Benefits Schedule (MBS)) for dementia assessment and diagnosis. Comprehensive cognitive assessment frequently requires multiple lengthy consultations involving cognitive testing, collateral history from family members, diagnostic discussions and care planning. These activities are not specifically recognized within current MBS arrangements, meaning dementia competes with other chronic disease activities that are better aligned with existing funding mechanisms [9]. Similar funding barriers have been identified in previous Australian studies, where inadequate remuneration has been recognized as a disincentive to timely dementia diagnosis and management [33]. Our findings extend this evidence by demonstrating that funding arrangements influence not only routine dementia care but also clinicians’ willingness and capacity to participate in quality improvement initiatives designed to improve dementia diagnosis. Improving dementia diagnosis in primary care is therefore likely to require both practice-level interventions and policy settings that better support the complexity of dementia care [37].

Implementation was also influenced by the role of PHNs as implementation partners. Although PHNs have established relationships with general practices and are well positioned to support quality improvement, their primary role is to commission services rather than directly deliver clinical programs [21]. Consequently, implementation competed with existing contractual obligations, organizational priorities and workforce capacity. Despite project funding being provided, participating PHNs reported that the Practice Change Program was not incorporated into routine operational budgets or supported by dedicated implementation staff. Communication challenges and differing expectations regarding implementation responsibilities further affected delivery. These findings align with the implementation science literature demonstrating that successful implementation depends on organizational readiness, clearly defined partner roles and shared ownership of implementation activities [35,36,38]. They also highlight challenges specific to implementing research through intermediary organizations such as PHNs, where research implementation must compete with existing contractual priorities and service obligations [39]. The evaluation also highlighted the importance of direct GP engagement. PHN staff reported that their routine interactions were more commonly with practice managers and practice nurses than with GPs, limiting opportunities to engage the clinicians primarily responsible for dementia diagnosis. Future implementation strategies should therefore include additional mechanisms to directly engage GPs alongside existing PHN practice facilitation activities.

### 4.2. Education Remains Necessary but Is Insufficient Alone

Consistent with international and Australian evidence, participating clinicians valued the education and reported improved confidence, communication and perceived capability [14,15,40,41].

Participating clinicians described the education as helpful in addressing misconceptions regarding the value of timely diagnosis and increasing their motivation to initiate conversations about cognitive change.

However, the findings also demonstrate the limitations of education as a stand-alone implementation strategy. High registration but relatively low attendance, together with limited overall reach, indicates that increasing knowledge alone is unlikely to achieve widespread changes in practice. These findings support evidence demonstrating that educational interventions improve clinician capability but rarely achieve sustained behaviour change unless combined with organizational support, implementation facilitation, audit and feedback, and workflow integration [15,16,42]. Education should therefore be considered one component of broader implementation strategies rather than the primary mechanism for achieving sustained practice change.

### 4.3. Audit and Benchmarking as a Catalyst for Practice Change

Audit and feedback are recognized implementation strategies capable of influencing professional behaviour [43]. One promising finding was clinicians’ response to tailored audit and benchmarking reports. Many participating GPs reported that they had been unaware of the discrepancy between documented dementia diagnoses within their practice and the expected prevalence based on their patient population. Presenting practices with locally relevant performance data increased awareness of potential underdiagnosis and stimulated reflection on opportunities for improving dementia identification. These findings are consistent with evidence that personalized audit and feedback are more likely to influence professional behaviour than generic educational messages because they make evidence–practice gaps directly visible to clinicians [44]. Our findings suggest this strategy may have particular value in dementia care, where clinicians may underestimate the extent of underdiagnosis within their own practices. Although the present study was not designed to determine whether benchmarking increased dementia diagnosis rates, these findings indicate that practice-level performance feedback may represent an important mechanism for supporting dementia quality improvement in primary care. Future research should investigate how benchmarking can be integrated into routine quality improvement programs and linked with ongoing practice facilitation.

### 4.4. Digital Decision Support Must Fit Routine Clinical Workflows

Although technically feasible, the dementia risk-alert tool achieved limited uptake because of software incompatibility, workflow disruption and concerns regarding usability and specificity. Furthermore, the intervention was not implemented exactly as intended because several variables included within the original CogDrisk algorithm [27] were unavailable in routinely collected electronic medical record data. Consequently, the implemented algorithm relied on a reduced set of variables, contributing to a large number of patients being identified as potentially eligible for dementia assessment. These findings are consistent with previous evaluations demonstrating that clinical decision-support tools are most effective when integrated seamlessly into routine clinical workflows and supported by reliable electronic health record data [45]. Our study extends this evidence by demonstrating that implementation fidelity may also be compromised when routinely collected data are insufficient to support the intended decision-support algorithm. Future refinement of the algorithm and greater interoperability across clinical software platforms will be important before wider implementation.

### 4.5. Lessons for Future Practice Change Interventions

Several practical lessons emerged from this evaluation. First, the intervention was perceived as acceptable among participating clinicians, although the limited representation of non-participating GPs constrains conclusions about acceptability across the broader target population. Second, implementation partnerships require clearly defined roles, realistic expectations and dedicated organizational resources. Third, audit and benchmarking achieved broad organizational reach and were perceived by participating clinicians as useful for identifying evidence–practice gaps and may provide a scalable quality improvement strategy. Fourth, digital decision-support tools should be developed using routinely available electronic medical record data and integrated into existing clinical workflows to maximize adoption. Finally, embedding process evaluation provided insights into differences in the reach, uptake, feasibility and perceived usefulness of individual intervention components, allowing refinement before wider implementation [22]. These lessons are consistent with contemporary implementation frameworks, which emphasize that successful implementation is determined by interactions between intervention characteristics, organizational context and implementation processes rather than by the intervention itself [23,46].

Beyond dementia care, the Facing Dementia Together study contributes to the complex interventions in the healthcare literature demonstrating that successful translation of evidence into routine primary care depends on addressing clinician capability, organizational readiness and health system context simultaneously [35]. High-quality educational resources alone are unlikely to produce widespread practice change unless implementation strategies also address workforce capacity, implementation partnerships, digital infrastructure and policy settings. Although undertaken in the context of dementia diagnosis, these implementation lessons are likely to be applicable to the introduction of other complex models of care for chronic conditions managed in primary care.

### 4.6. Strengths and Limitations

A strength of this study was the use of RE-AIM with Appropriateness to systematically examine multiple dimensions of implementation, drawing on quantitative and qualitative data from multiple sources. This approach provided insights into implementation processes, contextual influences and participant experiences that would not have been identified through outcome evaluation alone.

Several limitations should be acknowledged. Only three participants completed the two-month follow-up survey, substantially limiting assessment of sustained clinician-level Adoption and preventing adequate assessment of Maintenance. Although qualitative interviews provided examples of reported behaviour change and continued use of intervention components among participating clinicians, these data cannot establish the extent or sustainability of practice change across the broader target population.

Participation bias is also possible. Although purposive recruitment sought to include GPs who did not engage with the intervention, only one non-participating GP was interviewed. The qualitative findings therefore predominantly reflect the experiences of clinicians and stakeholders who engaged with the program, who may have been more motivated or held more favourable attitudes towards dementia care. Perspectives of non-participating clinicians are under-represented, and barriers to engagement within the broader target GP population may therefore not have been fully captured.

As intervention components were implemented concurrently as part of a multicomponent program, the evaluation cannot determine the relative contribution of individual components to reported changes or implementation outcomes. Furthermore, as this study evaluated implementation rather than effectiveness, it cannot determine whether the Practice Change Program improved dementia diagnosis rates or patient outcomes; these outcomes will be reported separately. Finally, the study was conducted across two Australian Primary Health Networks, which may limit transferability to other settings. However, many of the organizational and implementation challenges identified are consistent with those reported internationally for complex interventions in primary care, suggesting potential relevance beyond the study settings.

## 5. Conclusions

The Facing Dementia Together Practice Change Program was implemented across two Australian Primary Health Networks and was perceived as acceptable by participating clinicians, although engagement varied substantially across intervention components. Participating clinicians reported increased confidence following education and described practical resources and tailored audit and benchmarking data as useful for supporting dementia assessment and identifying potential evidence–practice gaps. The process evaluation identified organizational, workforce, digital infrastructure and implementation partnership factors that influenced intervention delivery and uptake.

These findings suggest that implementation of dementia practice-change initiatives requires more than clinician education alone and is influenced by organizational support, appropriate funding and policy settings, integrated digital infrastructure and adequately resourced implementation partnerships. Although undertaken in the context of dementia, these implementation lessons may have broader relevance for translating complex evidence-based interventions into routine primary care.

Future research should determine whether the Facing Dementia Together Practice Change Program improves dementia diagnosis and patient outcomes and identify effective approaches for supporting sustained implementation across diverse primary care settings.

## Figures and Tables

**Table 1:** Facing Dementia Together Practice Change Program process evaluation: RE-AIM with the addition of Appropriateness with description and data sources.

RE-AIM Criteria	Description	Data Sources	Data Sample
Reach	Number and characteristics of primary care staff (GPs, GPNs, managers, receptionists) reached and the way they were reached	Promotional activities and potential reach (A) Training activities run, enrolments in training, attendance at training by profession (A) Website interactions (B)	978 registrants 277 education attendees
Short-term Effectiveness (did knowledge and motivation change)	Increase in capability, opportunity, motivation of GPs and GPNs	Immediate post-training and 2-month post surveys (C1, C2) Stakeholder interviews (D)	143 survey respondents immediate post-training 3 survey respondents 2-month post-training 24 stakeholder interviews
Appropriateness (was content relevant and useful)	Relevance, suitability and usefulness of training, checklists and handouts, videos, risk popup, resource packs	Immediate post-training surveys (C1) Campaign team documentation (A) Team meeting minutes (E) Stakeholder interviews (D)	143 survey respondents immediate post-training 24 stakeholder interviews
Adoption (did GPs and GPNs try new behaviours)	Uptake of intervention components and clinician-reported changes in dementia identification and assessment-related behaviours	2-month post surveys (C2) Stakeholder interviews (D) Implementation records and analytics (A, B)	Implementation records and analytics 3 two-month follow-up survey respondents 24 stakeholder interviews
Implementation	Consistency and fidelity of training, whether the intervention components were delivered as planned	Campaign team documents (A) Team meeting minutes (E)	
Maintenance * (did GPs and GPNs continue with the behaviours, did partners continue with initiatives)	Evidence of sustained clinician behaviour change and continuation of implementation activities by partner organizations	Two-month follow-up survey (C2) Stakeholder interviews (D)	3 two-month follow-up survey respondents 24 stakeholder interviews

A = campaign team documents; B = website analytics; C1 = post-training survey; C2 = two-month post-training survey; D = stakeholder interviews; E = meeting team minutes. * Clinician-level Maintenance could not be adequately assessed because of insufficient longitudinal follow-up.

**Table 2:** Participants and characteristics by process evaluation activity.

Evaluation Activity/Characteristic	n/N (%)
Immediate post-training/activity survey *	
Online education	126/277 (45%)
Face-to-face education	16/16 (100%)
Lunchtime in-practice session	1/52 (2%)
Two-month follow-up survey (*N* = 3)	
Professional role	
General practitioner	2 (67%)
General practice nurse	1 (33%)
Gender	
Female	2 (67%)
Male	1 (33%)
PHN region	
Western Sydney PHN	2 (67%)
Western Victoria PHN	1 (33%)
Stakeholder interviews (N = 24)	
Professional role/involvement	
PHN staff (managers, practice facilitators and specialist teams)	11 (46%)
General practitioners engaged in the program	8 (33%)
General practice nurse engaged in the program	1 (4%)
General practitioner not engaged in the program	1 (4%)
Dementia Training Australia trainers (GPs)	3 (13%)
Gender	
Female	18 (75%)
Male	6 (25%)
Location	
Western Victoria PHN	17 (71%)
Western Sydney PHN	4 (17%)
Other	3 (13%)

Abbreviations: GP = general practitioner; PHN = Primary Health Network. * Immediate post-training/activity surveys were anonymous; professional role, gender and PHN region were not collected.

## Data Availability

The original contributions presented in this study are included in the article/Supplementary Materials. Further inquiries can be directed to the corresponding author.

## Supplementary Materials

The following supporting information can be downloaded at: https://www.mdpi.com/article/10.3390/ijerph23081086/s1, Table S1: Standards for Reporting Qualitative Research (SRQR).

## Abbreviations

The following abbreviations are used in this manuscript:

COM-B	Capability, Opportunity, Motivation-Behaviour
GP	General practitioner
GPN	General Practice Nurse
DTA	Dementia Training Australia
MBS	Medicare Benefits Schedule
PHN	Primary Health Network
RE-AIM	Reach, Implementation, Adoption, Implementation, Maintenance
SRQR	Standards for Reporting Qualitative Research
